# An extreme halophilic xylanase from camel rumen metagenome with elevated catalytic activity in high salt concentrations

**DOI:** 10.1186/s13568-019-0809-2

**Published:** 2019-06-17

**Authors:** Kamran Khalili Ghadikolaei, Elham Dasi Sangachini, Vasimeh Vahdatirad, Kambiz Akbari Noghabi, Hossein Shahbani Zahiri

**Affiliations:** Department of Energy and Environmental Biotechnology, National Institute of Genetic Engineering and Biotechnology (NIGEB), Tehran, Iran

**Keywords:** Xylanase, Halophilic, Extreme, Plant biomass

## Abstract

An extreme halophilic xylanase, designated as XylCMS, was characterized by cloning and expression of the encoding gene from a camel rumen metagenome. XylCMS proved to be a GH11 xylanase with high identity to a hypothetical glycosyl hydrolase from *Ruminococcus flavefaciens.* XylCMS with a molecular weight of about 47 kDa showed maximum activity at pH 6 and 55 °C. The enzyme activity was significantly stimulated by NaCl in 1–5 M concentrations. Interestingly, the optimum temperature was not influenced by NaCl but the *K*_cat_ of the enzyme was enhanced by 2.7-folds at 37 °C and 1.2-folds at 55 °C. The *K*_m_ value was decreased with NaCl by 4.3-folds at 37 °C and 3.7-folds at 55 °C resulting in a significant increase in catalytic efficiency (*K*_cat_/*K*_m_) by 11.5-folds at 37 °C and 4.4-folds at 55 °C. Thermodynamic analysis indicated that the activation energy (*E*_*a*_) and enthalpy (∆*H*) of the reaction were decreased with NaCl by 2.4 and threefold, respectively. From the observations and the results of fluorescence spectroscopy, it was concluded that NaCl at high concentrations improves both the flexibility and substrate affinity of XylCMS that are crucial for catalytic activity by influencing substrate binding, product release and the energy barriers of the reaction. XylCMS as an extreme halophilic xylanase with stimulated activity in artificial seawater and low water activity conditions has potentials for application in industrial biotechnology.

## Introduction

Xylanase is an important enzyme in the degradation of plant biomass by hydrolysing xylan as a main constituent of the plant cell wall. Xylan is composed of xylose monomers connected by β-1,4-glycoside linkages accounting for 30–35% of hardwoods, 15–30% of graminaceous plants, and 7–12% of gymnosperms. Xylanase has application in various industries where plant biomass hydrolysis is required for the production of human food, animal feed, paper, pulp, and biofuels (Adigüzel and Tunçer 2016; Kaur et al. 2016; Kumar et al. 2016; Yegin et al. 2018). Plant biomass as the most abundant renewable organic matter is regarded as a promising feedstock for sustainable production of green fuels. However, the recalcitrant structure of plant biomass is a major drawback in many industrial applications. Various physical, chemical, and enzymatic treatments have been developed for conversion of plant biomass into soluble fermentable sugars. Given the costs, safety, and environmental issues, enzymatic treatment has proved to be superior to other treatments. In general, enzymes with extremophilic characteristics are of special interest for industrial applications where harsh conditions such as high temperature, acidic or alkaline pH, and salt concentrations are required. Therefore, attempts have been underway to discover or develop novel xylanases with desired characteristics to meet the diversity of applications. In addition to the culture-dependent methods, metagenomics has been used as a powerful and culture-independent approach to mine various environments, such as the rumen of herbivores, for novel xylanases (Duan et al. 2009; Ghadikolaei et al. 2017, 2018; Hess et al. 2011; Xing et al. 2012). The metagenome obtained from environmental samples is a huge source of valuable genes from culturable and uncultured microorganisms. As an extremozyme, halophilic xylanase can be suitable for applications in low water activity conditions such as food industries and biorefineries. It is estimated that 1.9–5.9 m^3^ water is required for the production of 1 m^3^ biofuel from plant biomass (Fang et al. 2015). Therefore, the global scarcity of freshwater would be a drawback for plant biorefineries. In contrast, seawater is amply available on earth. Seawater has been shown to be an efficient solvent for hydrothermal pretreatment of plant biomass as well (Fang et al. 2015; Ren et al. 2016). In this study, extreme halophilic xylanase, designated as XylCMS, was discovered by cloning and recombinant expression of the encoding gene from a camel rumen metagenome, in *Escherichia coli*. The extreme halophilic xylanase not only was tolerant of salt but also its activity was stimulated in high salt concentrations. The mechanism of the salt stimulation was investigated using biochemical and biophysical methods. The halophilic xylanase may be interesting due to its potential application in high salt conditions.

## Materials and methods

### Chemicals and strains

The chemicals were generally purchased from Sigma-Aldrich (St. Louis, USA). Tryptone and yeast extract were from Merck (Darmstadt, Germany). Restriction enzymes, DNA marker, T4 DNA ligase, and DNA polymerase were purchased from Thermo Fisher Scientific (Waltham, USA). Protein marker was purchased from SinaClon (Tehran-Iran). The pET26b vector and *E. coli* strains including DH5α and BL21(DE3) were from Novagen (Madison, USA). The kits for PCR product purification and plasmid extraction were from GeneAll (Seoul, Korea). The Ni–NTA protein purification resin was purchased from Qiagen (Hilden, Germany).

### Bioinformatic analyses

The homology analysis was performed by Blast program at the NCBI website (https://blast.ncbi.nlm.nih.gov/Blast.cgi). The sequence alignment was conducted using Clustal Omega at EMBL-EBI (https://www.ebi.ac.uk/Tools/msa/clustalo/). The structural analyses were performed using SignalP-5.0 Server (http://www.cbs.dtu.dk/services/SignalP/) and the NCBI conserved domain database (CDD) (https://www.ncbi.nlm.nih.gov/Structure/cdd/wrpsb.cgi). The prediction of the enzyme structure was performed using RaptorX web server (http://raptorx.uchicago.edu/). The primer design, sequence analysis, and virtual cloning were performed using SnapGene software (GSL Biotech, http://snapgene.com).

### Recombinant protein production and purification

The xylanase-encoding gene (*xyl*-CMS) was obtained from a camel rumen metagenome which was available from a previous study (Gharechahi et al. 2015). The gene was isolated by PCR (polymerase chain reaction) using a pair of primers as forward (5′-AAGAATTCCAAGAATGGCATTTTAAAGAAAC-3′) and reverse (5′-AACTCGAGTTCGCCCTCAGCGC-3′) containing *Eco*RI and *Xho*I restriction sites, respectively. The PCR product was electrophoresed on a 1% agarose gel and purified by GeneAll kit. The gene was digested by *Eco*RI and *Xho*I and inserted using T4 DNA ligase into pET26b previously linearized by the same restriction enzymes. The resulting plasmid, pET*xyl*-CMS, was cloned in *E. coli* DH5α through chemical transformation and cultivation of the transformants on LB (Luria–Bertani) medium containing 50 µg/ml kanamycin. The recombinant plasmid was purified from the transformed *E. coli* DH5α cells using a GeneAll plasmid isolation kit and verified for authenticity by sequencing. For gene expression, pET*xyl*-CMS was used to transform *E. coli* BL21(DE3) and the transformants were grown on the LB/kanamycin medium in 37 °C, 200 rpm shaking conditions. The growing cells at an optical density (600 nm) of 0.8 were induced by 0.3 mM IPTG (Isopropyl β-d-1-thiogalactopyranoside) and then incubated for 24 h further in 25 °C, 120 rpm shaking conditions. The recombinant enzyme, Xyl-CMS, was isolated from periplasmic space and purified by NI–NTA (nickel nitrilotriacetic acid) resin using standard protocols from Qiagen. The purity and concentration of the enzyme were analyzed by SDS-PAGE (sodium dodecyl sulfate polyacrylamide gel electrophoresis) and Bradford method, respectively.

### Determination of xylanase activity

Xylanase activity was measured by quantification of reducing sugars released as a function of the enzyme reaction (Miller 1959). Activity assays were conducted in a reaction mixture composed of 90 µl of citrate buffer (pH 5) containing 1 mg/ml oat-spelt xylan and 10 µl of purified enzyme solution. All reactions were conducted for 10 min before being stopped by the addition of 3 volumes of DNS (3,5-dinitrosalicylic acid) reagent and heating in boiling water for 10 min. The light absorbance was measured at 540 nm to calculate the concentration of reducing sugars by a standard curve. One unit of enzyme activity was defined as the amount of enzyme required to produce one micromole of reducing sugars per minute under the assay conditions.

### Protein electrophoresis and zymography

The SDS-PAGE was conducted using a 12% polyacrylamide gel according to Laemmli method (Laemmli 1970). The native-PAGE was performed in a similar way except that SDS and β-mercaptoethanol were removed from the protocols for running buffer, polyacrylamide gel, and loading buffer preparation. In the preparation of the native polyacrylamide gel, 1% oat-spelt xylan was included and the electrophoresis was conducted at 4 °C for 2 h. In the end, the gel was washed with distilled water and incubated in citrate buffer (pH 5) at 37 °C for 1 h. Finally, the native gel was stained in 0.1% Congo Red solution for 30 min and then destained in 1 M NaCl solution.

### Effect of pH, temperature, and NaCl on enzyme activity

The effect of pH on enzyme activity was analyzed in different pH buffers including citrate buffer (pH 3–6), phosphate buffer (pH 6–8), and glycine–NaOH (pH 8–10) at 37 °C under standard conditions. The effect of temperature on enzyme activity was studied in citrate buffer (pH 5) at various temperatures in the range of 10–65 °C. The thermodynamic parameters were calculated using the Eyring’s absolute rate equation derived from the transition state theory (Eyring and Stearn 1939). The impact of high salt concentrations on the enzyme activity was studied by activity assays conducted at optimum pH and temperature in the presence of various NaCl concentrations (1–5 M). The activity of XylCMS was also studied in artificial seawater composed of NaCl, 26.29 g/l; KCl, 0.74 g/l; CaCl_2_, 0.99 g/l; MgCl_2_·6H_2_O, 6.09 g/l; MgSO_4_·7H_2_O, 3.94 g/l. The thermal stability of XylCMS was studied by determination of the residual activity in 10-min intervals during a 50-min incubation in citrate buffer (pH 5) at 50 °C, 55 °C, and 60 °C.

### Substrate specificity and kinetics

The substrate specificity of XylCMS was studied by activity assays conducted under standard conditions with different substrates including carboxymethyl cellulose (CMC), starch, and oat-spelt xylan (OSX) at 1% concentration. The Michaelis constant (*K*_m_) and maximum velocity (*V*_max_) of the enzyme for OSX hydrolysis at 37 °C and 55 °C were obtained by activity assays conducted with varying concentrations of the substrate in the range of 2–50 mg/ml in citrate buffer (pH 6). The effect of NaCl on the kinetic parameters was examined in two levels including 4 M and 3 M as optima for assays conducted at 37 °C and 55 °C, respectively. The scatter plot of enzyme activity versus substrate concentration was used to fit the data to the Michaelis equation by SigmaPlot software.

### Thin layer chromatography (TLC)

The products of oat-spelt xylan hydrolysis by XylCMS were analyzed by TLC. For this purpose, an enzyme reaction was conducted under standard conditions and, subsequently, 3 µl of the reaction solution was loaded on a TLC plate (silica gel 60 F_254_). The plate was allowed to dry at room temperature and then developed in a TLC tank using a mixture of 2-butanol, acetic acid, and water (2:1:1) as a mobile phase. In the end, the plate was dried at room temperature, sprayed with a mixture of ethanol and sulfuric acid (9:1) and baked at 110 °C for 10 min.

### Fluorescence spectroscopy

The effects of temperature, substrate, and NaCl on the enzyme conformation was studied by fluorescence spectroscopy using a Cary–Eclipse spectrofluorometer (Varian, Australia). For this purpose, the purified enzyme alone and also in the presence of NaCl, OSX or both was heated from 10 to 80 °C and the fluorescence emissions in the range of 300–500 nm were recorded at 10 °C intervals upon excitation at 280 nm. The spectra were corrected for the buffer contribution and the barycentric mean fluorescence (BCM) was calculated for each spectrum using the following equation:

$$ BCM\lambda = \frac{{\sum {\text{I}}\left( \lambda \right) \times \left( \lambda \right)}}{{\sum {\text{I}}\left( \lambda \right)}}, $$

where I(λ) is the fluorescence intensity at wavelength *λ*.

The first derivative of barycentric fluorescence plotted versus temperature was used to investigate the alterations in the melting pattern of XylCMS under the influence of OSX and NaCl. The melting temperature (T_m_) was estimated using the following relation:

$$ {{\text{T}}}_{{\text{m}}} = max\frac{\text{{dBCM}}}{{\text{dT}}} \left( {\text{T}} \right), $$

where *max* is the local maximum and dBCM/dT(T) is the first derivative of barycentric fluorescence as a function of temperature.

### GenBank accession number

The genes encoding for XylCMS was submitted to GenBank with the accession number of MG595703.1.

## Results

### Bioinformatic analysis

**Fig. 1:**
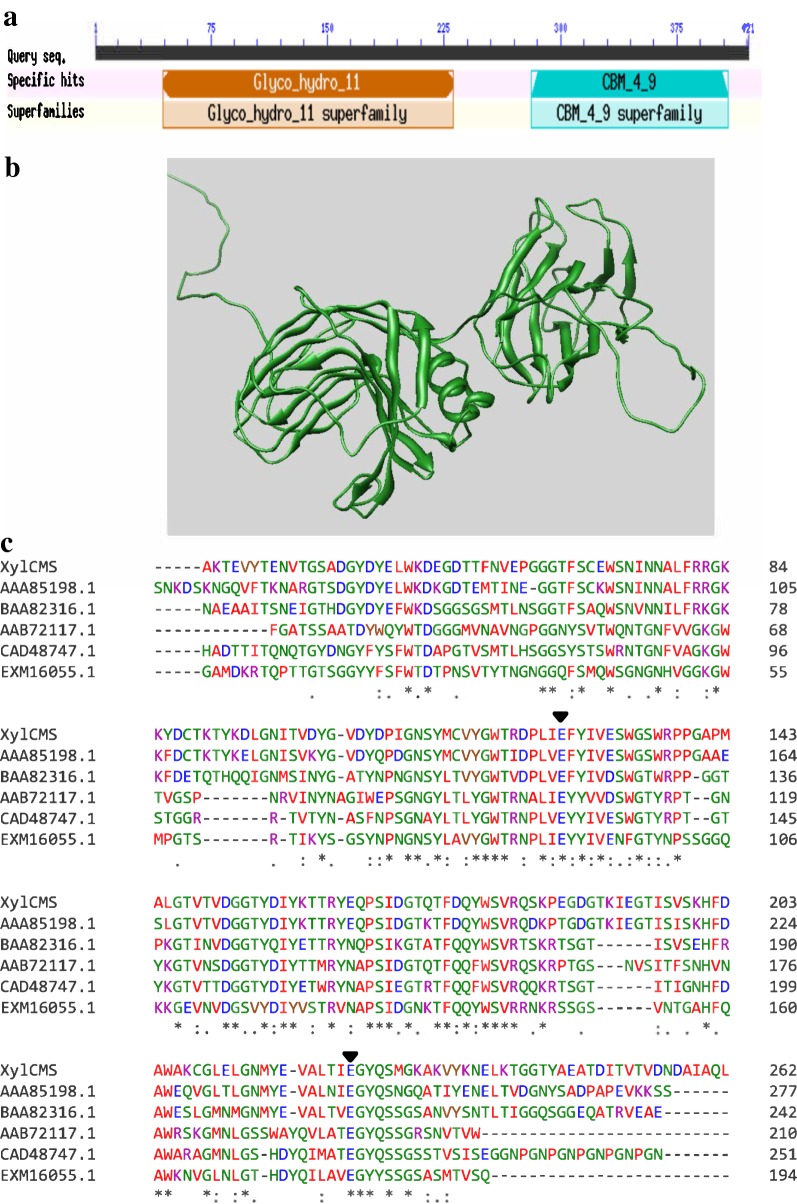
Structural analysis of XylCMS. **a** Conserved domains including a family 11 glycoside hydrolase and a carbohydrate binding module of family 4_9 were identified by NCBI’s conserved domain database. **b** The predicted tertiary structure of XylCMS confirming the modular structure of the enzyme. **c** Multiple sequence alignment of XylCMS with a few family 11 xylanases using Clustal Omega program. The identical and conserved residues are shown by stars and dots, respectively. The two glutamic acid catalytic residues are shown by arrowheads. The sequences are defined by their accession numbers at GenBank

A homology search using BlastP program at the NCBI database revealed that XylCMS exhibited 99% identity with a hypothetical protein of glycosyl hydrolase family 11 from *Ruminococcus flavefaciens*. However, the identity of XylCMS with already characterized xylanases was quite low, exhibiting most identity with XynA from *Ruminococcus albus* (59%), Xylanase 1 from a *Ruminococcus* sp. (46%), and XynB from *Ruminococcus flavefaciens* (44%). The structural analyses of XylCMS revealed the presence of a lipoprotein signal peptide (Sec/SPII), a xylanase domain, belonging to family 11 of glycoside hydrolases, and a carbohydrate binding module of family 4_9 (Fig. 1a). The homology modeling of XylCMS using RaptorX revealed a typical β-jelly roll structure for the xylanase domain and a β-sandwich structure for the CBM domain (Fig. 1b). The multiple sequence alignment of XylCMS with some other family 11 xylanases was used to identify conserved residues including two catalytic glutamic acids that are involved in substrate binding and catalysis (Fig. 1c).

### Heterologous protein expression and purification

**Fig. 2:**
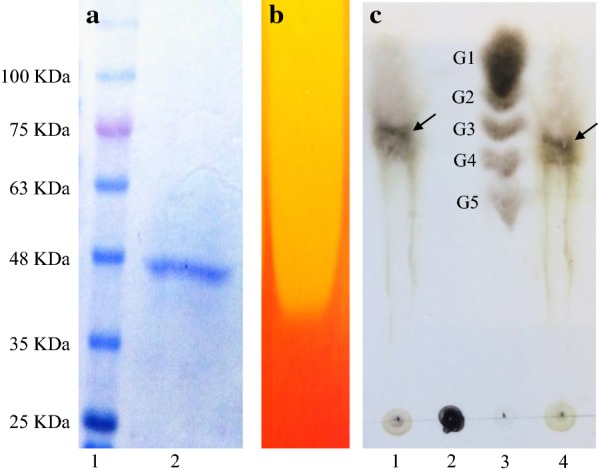
Analysis of purity, functional activity, and mode of action of XylCMS. **a** SDS-PAGE for purity analysis: Lane 1, protein molecular weight marker; Lane 2, purified XylCMS; **b** Zymogram prepared by Congo Red staining of a native PAGE gel indicating the functional activity of the purified enzyme as an unstrained area; **c** TLC analysis of OSX hydrolysis by XylCMS. Lane 1, enzyme-treated preparation; lane 2, control without enzyme; lane 3, standard molecular marker composed of mono-, di-, tri-, tetra-, and penta-glucose; lane 4, enzyme-treated preparation. The arrows indicate the hydrolysis products of OSX

The gene encoding XylCMS was cloned in pET-26b(+) in frame with the vector’s pelb sequence at N-terminus and the His-tag coding sequence at C-terminus. The resulting plasmid (pET-xylCMS) was successfully expressed in *E. coli* BL21(DE3) and the recombinant XylCMS was purified using Ni–NTA resin from the periplasmic cell fraction. The purity of the enzyme was confirmed by SDS-PAGE showing a single band of about 47 kDa which corresponded with the calculated molecular mass of the recombinant XylCMS (Fig. 2a). The zymography using a native-PAGE gel containing 1% OSX revealed that the purified enzyme was functionally active. The in-gel digestion of OSX was discernible as a yellowish area in a red background after staining with Congo Red (Fig. 2b). The products of OSX hydrolysis with XylCMS were analyzed by TLC. The result showed that XylCMS produced xylotetraose as the only hydrolysis product of OSX (Fig. 2c).

### Effects of pH and temperature on the enzyme activity

**Fig. 3:**
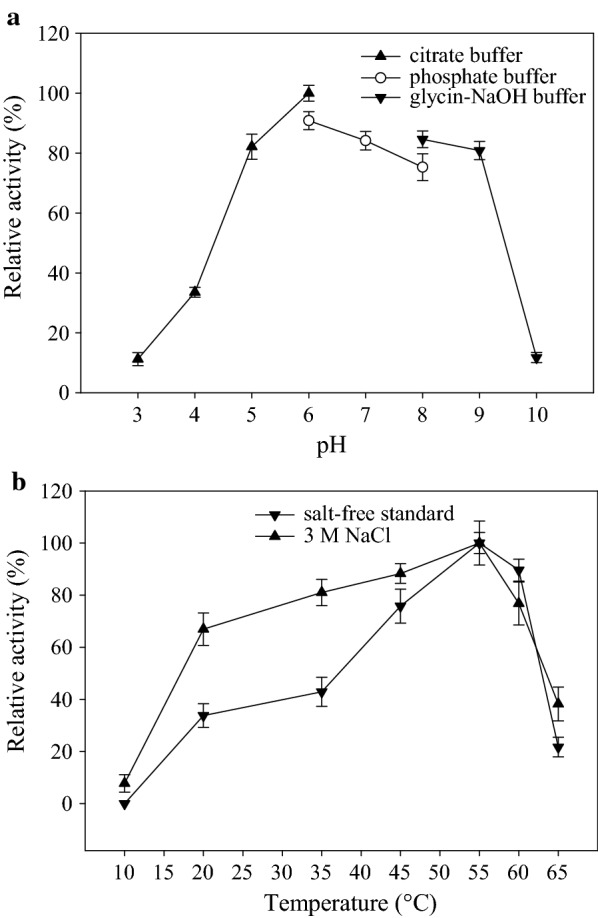
**a** Effect of pH on the activity of XylCMS. Activity assays were conducted in various pH buffers including citrate buffer for pH 3–6, phosphate buffer for pH 6–8, and glycine–NaOH buffer for pH 8–10 at 37 °C. The enzyme activities are shown as relative (%) to the highest value obtained at pH 6. **b** Effect of temperature on the activity of XylCMS. Activity assays were performed in citrate buffer (pH 6) at various temperatures under salt-free standard and 3 M NaCl conditions. The enzyme activities are shown as relative (%) to the highest values obtained at 55 °C

The effect of pH on the activity of XylCMS was studied in the range of pH 3–10. The results showed that the enzyme was active at all tested pH conditions with more than 80% activity over the pH range of 5 to 9, and at least 11% activity at other pH conditions (Fig. 3a)., The maximum activity of XylCMS was obtained at pH 6 in citrate buffer that was taken as the optimum pH condition for further activity assays. The profile of enzyme activity in the temperature range of 10–65 °C showed that the optimum temperature for XylCMS was 55 °C. The temperature profile of enzyme activity in 3 M NaCl revealed that the optimum temperature of XylCMS was not influenced by NaCl but the relative activity was significantly enhanced at temperatures below the optimum. The enzyme activity in 3 M NaCl was less temperature dependent and reached above 60% at a temperature below 20 °C while a similar activity in the absence of salt could be obtained at above 40 °C (Fig. 3b).

### Effects of NaCl on the enzyme activity and thermal stability

**Fig. 4:**
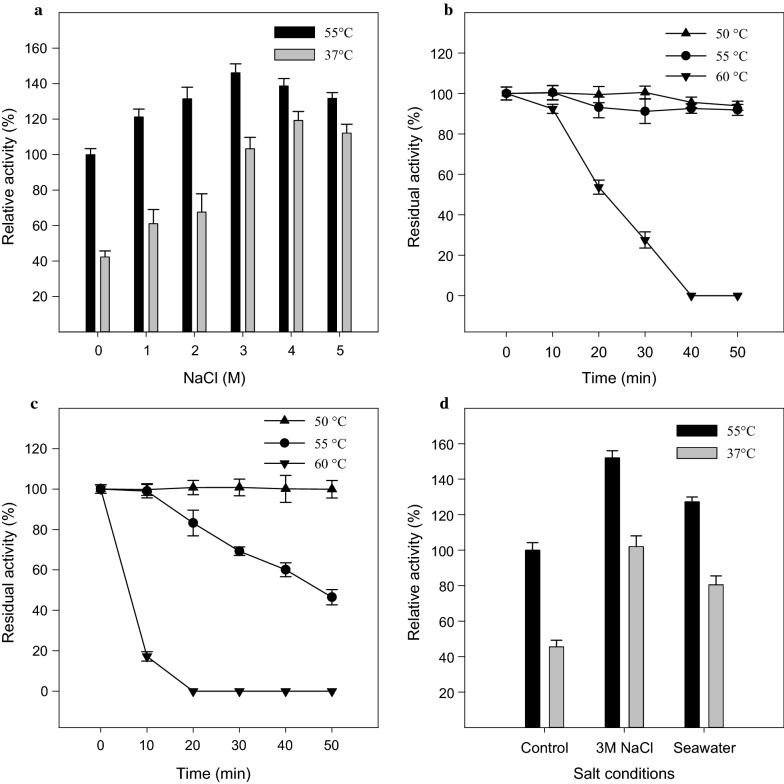
**a** Effect of NaCl on the activity of XylCMS. Activity assays were conducted in various NaCl concentrations in citrate buffer (pH 6) at 37 °C and 55 °C. The enzyme activities are shown as relative (%) to the enzyme activity in salt-free condition at 55 °C. **b** Thermal stability of XylCMS during 50 min incubation at 50 °C, 55 °C, and 60 °C in citrate buffer (pH 6). The residual activity was determined at 10 min intervals in citrate buffer (pH 6) at 55 °C and is shown as relative (%) to the enzyme activity before incubation. **c** Thermal stability of XylCMS under the influence of salt during 50 min incubation at 50 °C, 55 °C, and 60 °C in citrate buffer (pH 6) containing 3 M NaCl. The residual activity was determined at 10 min intervals in citrate buffer (pH 6) at 55 °C and is shown as relative (%) to the enzyme activity before incubation. **d** The catalytic activity of XylCMS in artificial seawater compared with that in the salt-free control condition and 3 M NaCl at 37 °C and 55 °C. The activities are presented as relative (%) to the enzyme activity in the salt-free condition at 55 °C

The effect of NaCl (1–5 M) on the activity of XylCMS was studied in citrate buffer (pH 6) at 55 °C as the optimum pH and temperature for the enzyme activity. The results showed that the activity of XylCMS was remarkably stimulated by NaCl in all tested concentrations. The highest activity with 46% enhancement as compared with control was obtained by 3 M NaCl. Interestingly, even at 5 M NaCl, the enzyme showed 31% higher activity compared to the NaCl-free control condition (Fig. 4a). The effect of NaCl on the enzyme activity was also assayed at 37 °C as the body temperature of camels. The results showed that the salt stimulation was even more striking at 37 °C giving rise to 2.4, 2.8, and 2.7 times enhancement of activity, respectively, in 3 M, 4 M, and 5 M NaCl concentrations as compared to the salt-free control condition at the same temperature (Fig. 4a). Therefore, the optimum NaCl concentration for maximum stimulation of the enzyme activity assayed at 55 °C and 37 °C was 3 M and 4 M, respectively. As Fig. 4a shows, the salt stimulation at 37 °C can enhance the enzyme activity to higher than the obtainable activity at 55 °C as the optimum temperature.

The thermal stability of XylCMS in NaCl-free condition as well as in 3 M NaCl was analyzed by determination of the residual activity of the enzyme in 10-minute intervals during a 50-min incubation at 50 °C, 55 °C, and 60 °C (Fig. 4b, c). The results showed that in the absence of NaCl, the enzyme with less than 8% loss of activity was almost stable at 50 °C and 55 °C. However, at 60 °C, its activity began to drop severely after 10 min and the enzyme completely inactivated during 40 min incubation. The analysis of thermostability in the presence of NaCl showed that the salt had a destabilizing effect on XylCMS. As a result, the enzyme activity began to decline after 10 min incubation at 55 °C and plunged to 47% during 50 min. Likewise, the enzyme in 3 M NaCl was significantly less stable at 60 °C with 83% loss of activity in 10 min and total inactivation in 20 min of incubation.

### Enzyme activity in artificial seawater

In order to investigate the potential application of XylCMS for catalysis in seawater, the enzyme activity was assayed in artificial seawater as the reaction medium. Figure 4d shows that XylCMS not only was functional in the artificial seawater but also its activity was stimulated by 27% at 55 °C and 77% at 37 °C as compared with corresponding salt-free controls.

### Fluorescence spectroscopy

**Fig. 5:**
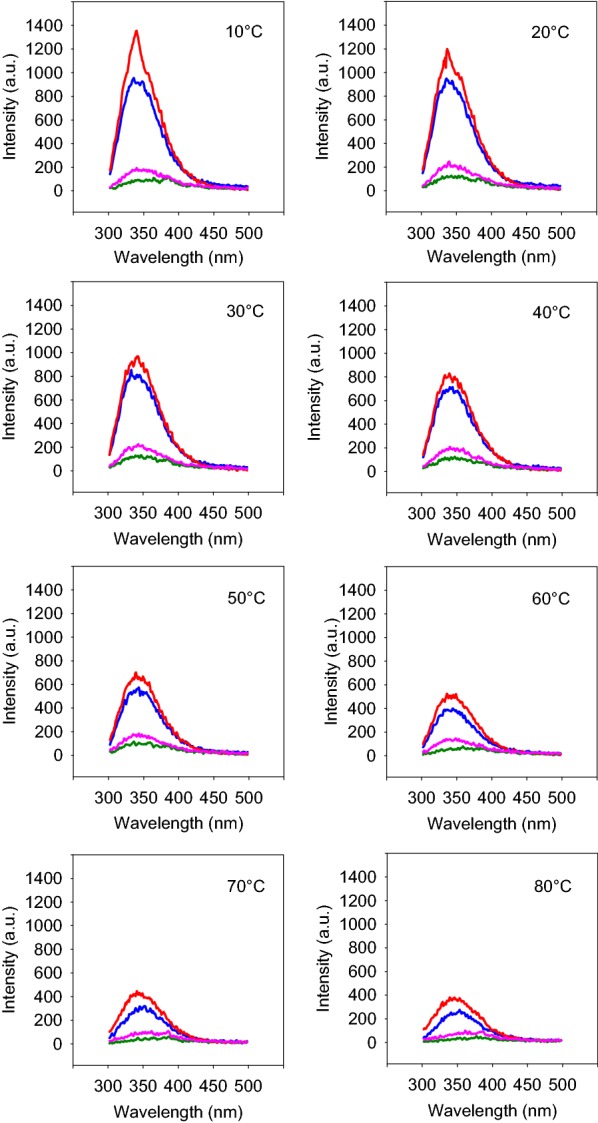
Analysis of intrinsic fluorescence of XylCMS alone and under the influence of 3 M NaCl and OSX at various temperatures in the range of 10–80 °C. At 10 °C intervals, the fluorescence intensity of the enzyme alone (blue line), with NaCl (red line), with OSX (green line), and in the presence of both NaCl and OSX (magenta line) was recorded in the range of 300–500 nm. At all temperatures, the fluorescence intensity of the enzyme was quenched with OSX but NaCl could improve the fluorescence activity of XylCMS both in the absence and presence of OSX

**Fig. 6:**
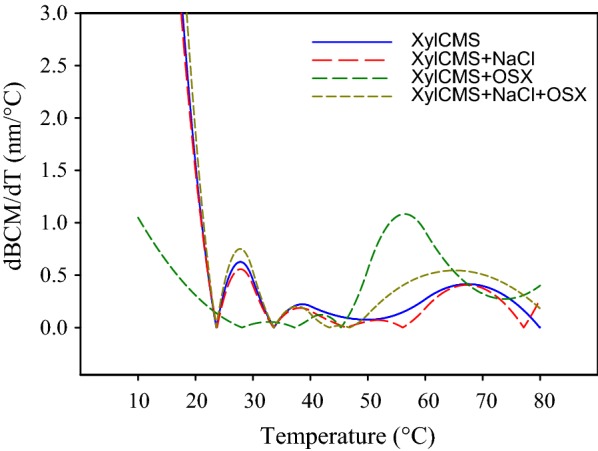
Influence of NaCl and OSX on the melting profile of XylCMS illustrated by plotting the first derivative of barycentric mean fluorescence as a function of temperature. The peak maximum indicates the melting temperature (T_m_) of the enzyme under various conditions

The impact of NaCl on the structure of XylCMS was investigated by fluorescence spectroscopy. For this purpose, the intrinsic fluorescence of the enzyme was analyzed alone and also in the presence of NaCl, OSX or both at various temperatures in the range of 10–80 °C. The results showed that the fluorescence intensity of XylCMS declined progressively with the increase of temperature and the intensity curve changed from a peak to plateau. In the presence of OSX as a substrate, the intrinsic fluorescence of the enzyme was severely quenched irrespective of temperature. However, NaCl (3 M) was shown to improve the fluorescence intensity of XylCMS at all tested temperatures and even in the presence of OSX, compared with the NaCl-free conditions (Fig. 5). The conformational changes of XylCMS as a function of temperature was investigated by the plot of dBCM/dT (nm/ °C) versus T (°C). The results showed that the melting profile of the enzyme was remarkably influenced by OSX. The melting temperature of XylCMS was estimated to be about 28 °C but in the presence of OSX, it was raised to 56 °C. In contrast, the melting profile of XylCMS was not much altered by 3 M NaCl and the melting temperature of the enzyme remained at about 28 °C in the presence of the salt. Interestingly, NaCl also counteracted the effects of OSX so that the melting temperature of XylCMS in the presence of both NaCl and OSX was still retained at 28 °C (Fig. 6).

### Substrate specificity and kinetics

The substrate specificity of XylCMS was studied using CMC, starch, and OSX as different substrates (1% w/v). The results showed that the enzyme could only hydrolyze OSX and no activity was detected on other substrates. Using activity assays with varying concentrations of OSX, the kinetic parameters of XylCMS were studied in the absence and presence of NaCl at both 37 °C and 55 °C (Table 1). The results showed that NaCl increased the *K*_cat_ of the enzyme by 2.7-folds at 37 °C and 1.2-folds at 55 °C. The *K*_m_ values were decreased with NaCl by 4.3-folds at 37 °C and 3.7-folds at 55 °C. The overall outcome was a significant increase in catalytic efficiency (*K*_cat_/*K*_m_) by 11.5-folds at 37 °C and 4.4-folds at 55 °C. The thermodynamic analysis of OSX hydrolysis by XylCMS indicated that NaCl decreased the activation energy (*E*_*a*_) of the reaction by 2.4-folds from 16.94 (kJ/mol) to 7.16 (kJ/mol). Likewise, the enthalpy (∆*H*) and Gibbs free energy (∆*G*) of the reaction decreased but the entropy (∆*S*) increased in the presence of NaCl (Table 2). The ∆*H*, in particular, was remarkably decreased more than threefold by NaCl (Table 2).

**Table 1:** Influence of NaCl on kinetic parameters of oat-spelt xylan hydrolysis by XylCMS at 37 °C and 55 °C

Conditions	Specific activity (U/mg)	*K*_m_ (mg/ml)	*K*_cat_ (1/s)	*K*_cat_/*K*_m_
37 °C (no NaCl)	345	24.4	214	8.77
37 °C (4 M NaCl)	740	5.7	579	101.6
55 °C (no NaCl)	1766	23.3	1383	59.4
55 °C (3 M NaCl)	2170	6.5	1700	261.5

**Table 2:** Influence of NaCl (3 M) on thermodynamic parameters of oat-spelt xylan hydrolysis by XylCMS at 37 °C and 55 °C

Conditions	∆*H* (kj/mol)	∆*G* (kj/mol)	∆*S* (kj/mol)	∆*G*_(E–S)_ (kj/mol)	∆*G*_(E–T)_ (kj/mol)
37 °C	14.36	62.22	− 0.154	7.62	− 6.19
37 °C + NaCl	4.58	61.25	− 0.182	5.54	− 9.24
55 °C	14.21	60	− 0.14	8.39	− 12.2
55 °C + NaCl	4.43	59.78	− 0.18	7.36	− 13.48

## Discussion

In this study, XylCMS was obtained from a camel rumen metagenome and characterized as a novel extreme halophilic xylanase. The blast search indicated that the gene encoding XylCMS should belong to *R. flavefaciens* due to the high identity of the enzyme with a hypothetical glycosyl hydrolase of the bacterium*. R. flavefaciens* is a strictly anaerobic coccus in the rumen that plays an important role in the decomposition of plant biomass. The ability of the bacterium to degrade plant polysaccharides is owing to a variety of lignocellulolytic enzymes that are secreted to the surroundings or anchor in the cell wall. Accordingly, a few xylanases have been characterized from *R. flavefaciens* (Flint et al. 1989, 1993; Zhang et al. 1994; Zhang and Flint 1992). However, XylCMS is distinguished from other characterized xylanases of the bacterium in that its activity is significantly stimulated by high salt concentrations. To the best of our knowledge, XylCMS is the first halophilic xylanase characterized from rumen. Even in comparison with other halophilic xylanases, XylCMS is noticeable for a significantly enhanced activity in a high NaCl concentration of 5 M (Table 3).

**Table 3:** Comparison of molecular weight, optimum pH, optimum temperature and activity at given NaCl concentrations between XylCMS and other characterized rumen and halophilic xylanases

Name	Source	Mol. weight^a^ (kDa)	Opt. pH^b^	Opt. temp.^c^ (°C)	Activity (%), salt con.^d^ (M)	References
Xyl-CMS	Camel rumen	46	6	55	132, 5	This study
Xyn10N18	Bovine rumen	54.5	6.5	35	ND	Gong et al. (2013)
XyIn-SH1	Holstein cattle rumen	39.5	6.5	40	ND	(Cheng et al. 2012)
XynGR40	Goat rumen	52.4	6.5	30	ND	Wang et al. (2011)
Xyn-lxy	Hu sheep rumen	71.3	6	50	ND	Wang et al. (2015)
ND^e^	*Bacillus pumilus*	39.6	8	40	73, 2.6	Menon et al. (2010)
XynSL3	*Alkalibacterium* sp.	150	9	55	60, 3	Wang et al. (2017)
*XynFCB*	*Thermoanaerobacterium saccharolyticum*	50	6.4	63	67, 2.6	Hung et al. (2011)
*XynA*	*Zunongwangia profunda*	43.7	6.5	30	100, 5	Liu et al. (2014)
ND	*Aureobasidium pullulans*	21.6	4	30–50	100, 3.4	Yegin (2017)
ND	*Chromohalobacter* sp.	15	9	65	90, 5	Prakash et al. (2012)
XynRBM26	*Massilia* sp. RBM26	45	5.5	45	86, 5	Xu et al. (2016)
Excg1	*Colletotrichum graminicola*	20	5.5	65	50, 3	Carli et al. (2016)

^a^Molecular weight

^b^Optimum pH

^c^Optimum temperature

^d^Salt concentration

^e^Not determined

For instance, in the case of two extremely halotolerant xylanases of a halophilic bacterium, the maximum activity was obtained with 1 M salt but the enzyme activity was inhibited in higher salt concentrations (Wejse et al. 2003). In another study on a haloalkaline xylanase from *Bacillus pumilus* GESF-1, the enzyme activity was enhanced by 60% in the presence of 2.5% NaCl (less than 0.5 M) but inhibited in higher salt concentrations (Menon et al. 2010). The activity of halotolerant xylanase from *Massilia* sp. RBM26 has been reported to be stable in NaCl concentrations of up to 1.5 M but at higher salt concentrations the activity decreased steadily to 85% in 5 M NaCl (Xu et al. 2016). The salt-stable alkaline xylanase of *Alkalibacterium* sp. SL3 has been shown to lose activity progressively with the increase of NaCl concentration in the range of 0.25–4.5 M (Wang et al. 2017). In contrast, XylCMS was proved in the current study to be an extreme halophilic xylanase with elevated activity in high NaCl concentrations up to 5 M that is unprecedented. It was revealed by fluorescence spectroscopy that XylCMS in 3 M NaCl exhibits more fluorescence activity during thermal denaturation. In other words, although the enzyme follows a general trend of thermal denaturation, its fluorescence activity tends to be higher in the presence of salt. The analysis of barycentric fluorescence indicated that the melting temperature of XylCMS was not altered by NaCl but raised from 28 °C to about 56 °C by OSX as substrate. It means that XylCMS gains more stability by the formation of the enzyme–substrate (ES) complex. In contrast, NaCl seems to exert a destabilizing effect on the enzyme conformation in the ES complex as was indicated by the restoration of the melting temperature to about 28 °C. The effect of NaCl gives rise to a more flexible structure of the enzyme that has less thermal stability as compared with the salt-free condition. The destabilizing effect of NaCl was also corroborated by the experimental study of thermal deactivation indicating that XylCMS deactivated more rapidly in the presence of NaCl. The *E*_*a*_ and ∆*H* of XylCMS were substantially decreased in the presence of NaCl indicating that the reaction has lowered energy requirements and, therefore, is more efficient under the influence of salt. In addition, a lower ∆*H* means that the reaction is less temperature dependent resulting in elevated rates of catalytic activity at temperatures below the optimum temperature. From the findings, it can be speculated that XylCMS is tolerant of high salt concentrations because of its high intrinsic flexibility. However, in the presence of substrate, the enzyme becomes less flexible possibly due to the formation of new inter- and intramolecular interactions. It seems that NaCl by eliminating the new interactions can restore the flexibility of the enzyme. The enzyme flexibility is crucial for catalytic activity by influencing substrate binding, product release and the energy barriers of the catalyzed reaction (Hammes-Schiffer and Benkovic 2006). On the other hand, the substrate affinity of XylCMS improves in the presence of salt as was indicated by a significant decrease in *K*_m_ value. Therefore, it seems that NaCl exerts three distinct effects on XylCMS: (1) a delicate stabilizing effect in certain locations by which the enzyme becomes partially structured as indicated by its elevated intrinsic fluorescence intensity; (2) an overall destabilizing effect that leads to more flexibility but less thermal stability of the enzyme in the ES complex as revealed by the barycentric fluorescence and the activity-stability analysis; (3) improvement of the affinity of the enzyme to substrate.

For industrial applications, extremophilic enzymes such as halophilic xylanases are more practical than normal enzymes to cope with the harsh conditions that are required or desired in different processes (Chen and Jiang 2018). An important challenge of industrial biotechnology is the high consumption of fresh water. A practical solution is the replacement of fresh water with abundant seawater in water-intensive industries such as lignocellulosic biorefineries. In this context, a hlophilic xylanase has potential application for enzymatic decomposition of plant biomass in seawater. In the other hand, plant biomass has a complex and tough structure that resists enzymatic hydrolysis. It has been shown that pretreatment with NaCl is a cost-effective, green method that can significantly improve the efficiency of enzymatic hydrolysis of plant biomass (Jiang et al. 2015). Therefore, XylCMS as a halophilic xylanase may be useful in future applications in the production of commodity chemicals such as biofuels from plant biomass. The halophilic xylanase has also potential application in bakery, pulp and paper industries due to the ability of the enzyme to function under low water activity conditions.

## Data Availability

Not applicable.

## Abbreviations

BCM: barycentric mean fluorescence
CDD: conserved domain database
CMC: carboxymethyl cellulose
DNA: deoxyribonucleic acid
DNS: 3,5-dinitrosalicylic acid
*E. coli*: *Escherichia coli*
*E*_*a*_: activation energy
EMBL-EBI: European Molecular Biology Laboratory-European Bioinformatics Institute
GH11: glycosyl hydrolase family 11
IPTG: isopropyl β-d-1-thiogalactopyranoside
*K*_cat_: catalytic constant
*K*_m_: Michaelis constant
LB: Luria–Bertani
ml: millilitre
NCBI: National Center for Biotechnology Information
NI–NTA: nickel nitrilotriacetic acid
OSX: oat-spelt xylan
PCR: polymerase chain reaction
*R. flavefaciens*: *Ruminococcus flavefaciens*
SDS-PAGE: sodium dodecyl sulfate polyacrylamide gel electrophoresis
TLC: thin layer chromatography
T_m_: melting temperature
µg: microgram
